# Creb1 regulates late stage mammalian lung development via respiratory epithelial and mesenchymal-independent mechanisms

**DOI:** 10.1038/srep25569

**Published:** 2016-05-06

**Authors:** N. Antony, A. R. McDougall, T. Mantamadiotis, T. J. Cole, A. D. Bird

**Affiliations:** 1 Department of Biochemistry & Molecular Biology, Monash University, Clayton, 3800, Victoria, Australia; 2 The Hudson Institute of Medical Research, Clayton, 3168, Victoria, Australia; 3 Department of Pathology, University of Melbourne, Parkville, 3010, Victoria, Australia

## Abstract

During mammalian lung development, the morphological transition from respiratory tree branching morphogenesis to a predominantly saccular architecture, capable of air-breathing at birth, is dependent on physical forces as well as molecular signaling by a range of transcription factors including the cAMP response element binding protein 1 (Creb1). *Creb1*^−/−^ mutant mice exhibit complete neonatal lethality consistent with a lack of lung maturation beyond the branching phase. To further define its role in the developing mouse lung, we deleted *Creb1* separately in the respiratory epithelium and mesenchyme. Surprisingly, we found no evidence of a morphological lung defect nor compromised neonatal survival in either conditional Creb1 mutant. Interestingly however, loss of mesenchymal *Creb1* on a genetic background lacking the related *Crem* protein showed normal lung development but poor neonatal survival. To investigate the underlying requirement for Creb1 for normal lung development, *Creb1*^−/−^ mice were re-examined for defects in both respiratory muscles and glucocorticoid hormone signaling, which are also required for late stage lung maturation. However, these systems appeared normal in *Creb1*^−/−^ mice. Together our results suggest that the requirement of Creb1 for normal mammalian lung morphogenesis is not dependent upon its expression in lung epithelium or mesenchyme, nor its role in musculoskeletal development.

Development of the mammalian lung is a highly intricate, multiphase process which is regulated largely by inter-germ layer molecular signaling, and by physical forces. Initially, epithelial buds emerge from the foregut endoderm, then branch extensively into the surrounding mesenchyme to produce the respiratory tree. In mice, these events begin during the embryonic phase (~E9.5-E10.5) and are essentially complete by the end of the “pseudoglandular” phase (~E10.5-E16.5)^1^. Subsequently, during the “cannalicular” (~16.5-E17.5) and “saccular” (~E17.5 till postnatal day 0.5) phases the mesenchymal content is considerably reduced and un-expanded distal epithelial tubules undergo extensive remodeling to form a fluid-filled dilated structure, with clefts (or primary septae) emerging to subdivide terminal airways into primitive alveolar sacs^2^. Concurrently, epithelial progenitors within distal tubules differentiate into type-I and –II alveolar epithelial cells (AECs) which then populate these sacs, mediating gas exchange and surfactant biosynthesis, respectively. To survive birth, which in rodents occurs during the saccular phase, proper sacculation is critical to ensure sufficient oxygen delivery to the blood once in a gaseous environment^1^. Although numerous studies have elegantly described the complex signaling pathways which regulate early lung budding and branching events, the molecular mechanisms which mediate the morphological transition from a pseudoglandular to a saccular structure are poorly understood.

Among the few factors implicated to have an important function during the lung saccular stage is the cAMP response element binding protein (Creb1). Creb1 is a member of the Creb/Atf subfamily of cAMP-responsive basic region-leucine zipper (bZIP) transcription factors which includes the cAMP response element modulatory protein (Crem) and activating transcription factor 1 (Atf1)^3^. In the nucleus, Creb1 is bound to DNA at specific gene promoter regions termed cAMP response elements (CREs) and transactivation normally only occurs when Creb1 is activated by upstream Ser/Thr-kinases, including cAMP-dependent PKA, which phosphorylate Ser-133^4^. Due to a high similarity in transactivation domain and bZIP (DNA binding domain) sequence identity between Creb1, Crem and Atf1, all can be activated by the same kinases and bind to target gene promoters as homodimers or as heterodimers with each other, thus providing a complex level of gene regulatory diversity^5^.

The role of Creb1/Atf family members in developmental (and other) processes has, in part, been revealed by gene targeted mutations in mice. Inactivation of *Atf1* or *Crem* does not appear to affect gross development, although *Crem*^−/−^ males become sterile due to impaired spermatogenesis^6^,^7^. Mice lacking *Creb1* isoforms α and β (*Creb1*^αΔ^) are found at a reduced Mendelian frequency indicating a developmental disadvantage, but are otherwise viable and healthy^8^. However, complete inactivation of Creb1 function via loss of isoforms α, β and Δ (*Creb1*^−/−^) suffer multiple defects including reduced birth weight, brain deformities, impaired T cell development and cessation of lung sacculation causing neonatal lethality^9^. Our investigation of a similar *Creb1* mutant revealed an almost identical whole body and respiratory phenotype, and further showed that *Creb1*^−/−^ lungs had not developed beyond the pseudoglandular stage^10^. Importantly, analysis of this latter mutant demonstrated that complete loss of Creb1 could not be rescued by the activities of other Creb/Atf members, a compensatory mechanism described in studies using both compound *Creb*/*Atf1* knockouts^7^ as well as conditional *Creb1* mutants on a genetic background lacking either *Crem* or *Atf1*^11^,^12^.

Although the phenotype of total *Creb1*-mutants indicates a requirement for Creb1 signaling during lung saccular stage development, the underlying molecular mechanisms remain unclear. Localization of transcriptionally active Creb1 in the fetal mouse lung using Ser133 phosphorylated Creb1 (pCreb1) immunostaining strongly suggests a role in the distal epithelium^10^,^13^. This is also consistent with the known stimulatory effect of cAMP on surfactant protein family gene transcription (Reviewed in^14^). Furthermore, total *Creb1*-mutants display a severe lack of AEC, and to a lesser extent, proximal epithelial cell marker expression while mesenchymal-derived vasculature and smooth muscle development appear unaffected^10^. Despite this, persistent Creb1 and pCreb1 expression in mesenchymal cell subsets, particularly during the saccular phase^10^, also suggest a function for Creb1 in the developing lung mesenchyme.

In this study, we have investigated the functional role of Creb1 in the developing respiratory epithelium and mesenchyme by conditional Creb1 deletion within these germ layers. We initially explored the importance of Creb1 for neonatal survival, morphological lung development and also epithelial cell differentiation during the saccular phase. Surprisingly, in these analyses we did not observe any requirement for Creb1 in any specific lung germ layer. To test for possible compensation by Creb/Atf members in conditional Creb1 knockout lungs, we assessed relative *Crem* and *Atf1* gene expression levels and also lung morphology in conditional knockouts on a *Crem* deficient background. Ruling out these effects, we then re-examined total *Creb1*^−/−^ fetal mice for defects in extra-pulmonary systems which are known to indirectly influence saccular lung development. Together our results indicate that loss of either respiratory epithelial or mesenchymal Creb1 expression is dispensable for lung development and neonatal survival, and is not compensated for by other Creb/Atf1 members. Lastly, we find that the respiratory phenotype in *Creb1*^−/−^ mice is likely not due to deficiencies in either glucocorticoid (GC) signaling or muscular-driven physical forces which are also essential for normal saccular lung development.

## Results

### Conditional loss of Creb1 in lung epithelium and mesenchyme does not impair survival at birth

**Figure 1:**
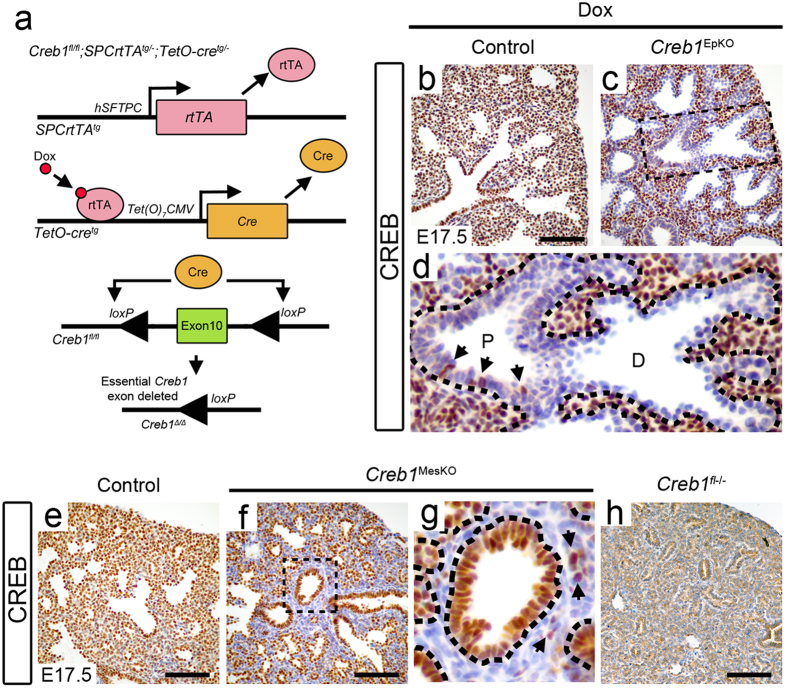
*Creb1* deletion in developing lung epithelium and mesenchyme. (**a**) Mechanism of respiratory-epithelial *Creb1* deletion using a doxycyline-inducible triple transgenic mouse system. In the presence of *Creb1*^*fl*^, *SPCrtTA*^*tg*^ and *TetO-cre*^*tg*^ alleles, doxycycline treatment induces lung-epithelial Cre recombinase expression. (**b**–**d**) Immunohistochemistry for Creb1 in Dox-treated E17.5 control (*Creb1*^*fl/fl*^; *SPCrtTA*^*tg/*−^) and *Creb1*^EpKO^ lungs. Creb1 expression is virtually ubiquitous in control lungs (**b**) while lung epithelial Creb1 expression is mostly absent in *Creb1*^EpKO^ lungs (**c**). Boxed area of (**c**) is magnified in (**d**) to show sporadic Creb1+ cells (arrows) in the *Creb1*^EpKO^ proximal lung epithelium. (**e**–**h**) Creb1 immunohistochemistry in E17.5 (non Dox-treated) control, *Creb1*^MesKO^, and *Creb1*^*fl*−/−^ lungs. Control lungs show almost ubiquitous Creb1 expression (**e**) while mesenchymal Creb1 expression is mostly lost in *Creb1*^MesKO^ lungs (**f**). Boxed area of (**f**) is magnified in (**g**) to show rare Creb1+ cells (arrows) in the *Creb1*^MesKO^ lung mesenchyme. Creb1 expression is completely absent in *Creb1*^*fl*−/−^ lungs (**h**). Dotted lines in (D) and (G) indicate the epithelial-mesenchymal boundary. ‘P’ indicates proximal airway lumen; ‘D’ indicates distal airway lumen. All images are representative of at least three animals per genotype. Scale bars: *b,c,e,f,h*; 90 μm.

We first assessed the impact of lung epithelial Creb1 deletion on neonatal survival. Lung epithelial deletion was achieved using a doxycycline-inducible Cre/LoxP system. In this approach, *Creb1*^*fl/fl*^ mice were bred to *SPCrtTA*^*tg/*−^ and *TetO-cre*^*tg/*−^ strains to generate triple transgenic pups bearing all three alleles. This allows for *Creb1* deletion in the respiratory epithelium due to the restricted expression of reverse tetracycline transactivator (rtTA) from the *hSFTPC* promoter. The rtTA, together with externally provided doxycycline in rodent food, then drive expression of Cre recombinase via the *Tet*(*O*_*7*_)*CMV* promoter to completely remove Creb1 expression in the lung epithelium (Fig. 1a). Therefore, maternal doxycycline-fed triple transgenic mice (*Creb1*^*fl/fl*^; *SPCrtTA*^*tg/*−^; *TetO-cre*^*tg/*−^) are hereafter referred to as ‘*Creb1*^EpKO^’ mice. To account for known deleterious effects associated with doxycycline and rtTA toxicity in embryonic mouse lungs^15^, *Creb1*^*fl/fl*^; *SPCrtTA*^*tg/*−^ animals were used as controls for *Creb1*^EpKO^ mice in all analyses. Surprisingly, genotyping analysis of litters at two weeks of age showed no significant deviations from the expected 25% Mendelian frequency in *Creb1*^EpKO^ mice (Table 1). Furthermore, we did not observe any obvious abnormalities in these mice throughout adulthood.

We then investigated the requirement for mesenchymal Creb1 in the developing lung via mesenchymal-specific *Creb1* deletion. This was achieved using a Dermo1-cre strain which express Cre recombinase as early as E13.5 within a variety of mesodermal-derived tissues including lung mesenchyme^16^,^17^,^18^. However, due to the very close proximity of the *Creb1* and Dermo1 (*Twist2*) genes on chromosome 1 (~29 Mb apart), matings between *Creb1*^*fl/+*^;*Dermo1*^*Cre/*+^ and *Creb1*^*fl/fl*^ mice resulted in a highly unequal frequency of progeny genotypes, (distinct from the knockout phenotype) and was therefore not an appropriate means of assessing survival. Nevertheless, the few *Creb1*^*fl/fl*^;*Dermo1*^*Cre/*+^ mice (hereafter referred to as *Creb1*^MesKO^) that were produced appeared healthy and were fertile. In addition, when *Creb1*^MesKO^ mice were mated with *Creb1*^*fl/fl*^ mice, we found no significant deviations from the expected 50% *Creb1*^MesKO^ frequency when litters were genotyped at two weeks of age (Table 2). We also investigated Creb1 deletion in the endothelium using *Tie2*-cre mice which express Cre recombinase as early as E7.5 in the vascular endothelium^19^. However, in the two litters analysed (15 pups) we did not observe any loss of survival or other defects in *Creb1*^*fl/fl*^*;Tie2*^*Cre/*+^ mice (hereafter referred to as “*Creb1*^EndoKO^”). Total *Creb1* knockout mice were also generated by mating *Creb1*^*fl/fl*^ to CMV-Cre mice, which show Cre activity in all tissues before embryo implantation^20^. Complete neonatal lethality in a separate strain of *Creb1*^−/−^ mice (bearing an exon 10 disruption) has previously been shown^9^, and although our *Creb1*^*fl/fl*^;*CMV*^*Cre/*+^ mice (hereafter referred to as “*Creb1*^*fl*−/−^”) represent a slightly different *Creb1* mutation, we have previously demonstrated the same morphological (whole body dwarfism) and respiratory phenotype (loss of sacculation) in the mice described herein^10^, as that exhibited in the previous strain. Therefore, we assumed an outcome of neonatal lethality in *Creb1*^*fl*−/−^ mice and we did not investigate this further.

### Efficacy of respiratory epithelial and mesenchymal Creb1 deletion

To determine the degree of Creb1 deletion in respective lung germ layers, we performed immunohistochemistry for Creb1 at E17.5 where the mesenchymal tissue between developing epithelial buds is clearly defined. In all controls examined, Creb1 expression was highly expressed in all germ layers (Fig. 1b,e). Respiratory epithelial-specific Creb1 deletion was variable in *Creb1*^EpKO^ mice, with some animals showing almost complete loss of Creb1 expression in lung epithelium (as shown in Fig. 1c,d) while others showed greatly reduced, but incomplete deletion in lung epithelium. The extent of Creb1 deletion in *Creb1*^EpKO^ mice appeared particularly variable in the proximal lung epithelium, with distinct clusters of Creb1+ cells often observed in the conducting airway (Supplementary Fig. S1). This was also evident as early as E15.5, although at these earlier stages Creb1+ cells were less frequently observed (Supplementary Fig. S1). In comparison, the distal lung epithelium showed a much more complete and consistent absence of Creb1 expression in *Creb1*^EpKO^ mice (Fig. 1d and Supplementary Fig. S1). In *Creb1*^MesKO^ mice, lung mesenchymal Creb1 expression was almost completely abolished, although a few rare interstitial Creb1+ cells were observed (Fig. 1f,g). We also observed loss of Creb1 in chondrocytes in *Creb1*^MesKO^ mice, consistent with the known spatial expression of *Dermo1*^*Cre*^ in several non-respiratory tissues^16^ (Supplementary Fig. S1). Efficient Creb1 deletion in endothelial cells was also observed in lungs of *Creb1*^EndoKO^ mice (Supplementary Fig. S1). As expected, total loss of Creb1 expression was always observed in the lung of *Creb1*^*fl*−/−^ mice (Fig. 1h).

### The Creb1^−/−^ respiratory phenotype is not recapitulated by loss of Creb1 in lung epithelium and mesenchyme

**Figure 2:**
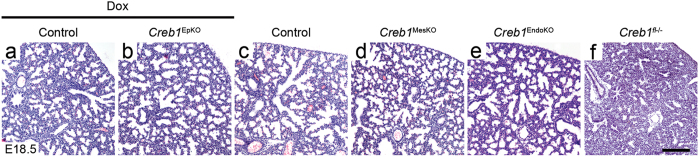
Normal lung development in *Creb1*^EpKO^, *Creb1*^MesKO^ and *Creb1*^EndoKO^ mice. (**a**–**f**) Haematoxylin and eosin-stained tissue sections from E18.5 *Creb1*^EpKO^, *Creb1*^MesKO^, *Creb1*^EndoKO^ and *Creb1*^*fl*−/−^ lungs. Loss of Creb1 separately in lung epithelium (**b**), mesenchyme (**d**) and endothelium (**e**) produces no phenotype compared to controls (**a**,**c**). Lungs of *Creb1*^*fl*−/−^ lungs mice exhibit a severe defect in sacculation, with little or no expansion of proximal and distal airways (**f**). All images are representative of at least three animals per genotype. Scale bar: 180 μm.

Histological analysis was used to investigate potential alterations to lung morphology in E18.5 *Creb1*^EpKO^, *Creb1*^MesKO^ and *Creb1*^EndoKO^ mice. Unexpectedly, we found no consistent morphological differences in E18.5 *Creb1*^EpKO^ lungs when compared to controls (Fig. 2a,b). As we had often found variable lung epithelial Creb1 deletion in fetal *Creb1*^EpKO^ mice, we looked to see whether the extent of Creb1 deletion correlated to any degree of abnormal lung morphology. However, even in *Creb1*^EpKO^ mice with the most complete level of lung epithelial Creb1 deletion we noted no difference in morphology compared with littermate controls. A tissue-to-airspace measurement also revealed no difference in overall cellularity in E17.5 *Creb1*^EpKO^ lungs (Supplementary Fig. S2). Similarly, loss of mesenchymal or endothelial Creb1 in *Creb1*^MesKO^ and *Creb1*^EndoKO^ mice respectively, did not affect lung morphology (Fig. 2c–e). Due to the lack of significant pCreb1 expression in lung endothelial cells relative to other mesenchymal lineages and the epithelium^10^, from herein our analysis does not include *Creb1*^EndoKO^ mice. Adult lung structure in *Creb1*^EpKO^ and *Creb1*^MesKO^ mice also appeared similar to controls (Supplementary Fig. S3). In comparison, lungs of E18.5 *Creb1*^*fl*−/−^ mice displayed almost no expansion of airways and retained a morphology similar to that of late pseudoglandular-stage lungs, consistent with previous analysis of the *Creb1*^−/−^ respiratory phenotype^10^ (Fig. 2f). Together these results suggest that Creb1 expression in lung epithelium, mesenchyme or endothelium separately is not required for normal lung development.

### Epithelial differentiation is unaffected in *Creb1* ^EpKO^ and *Creb1* ^MesKO^ fetal lungs

**Figure 3:**
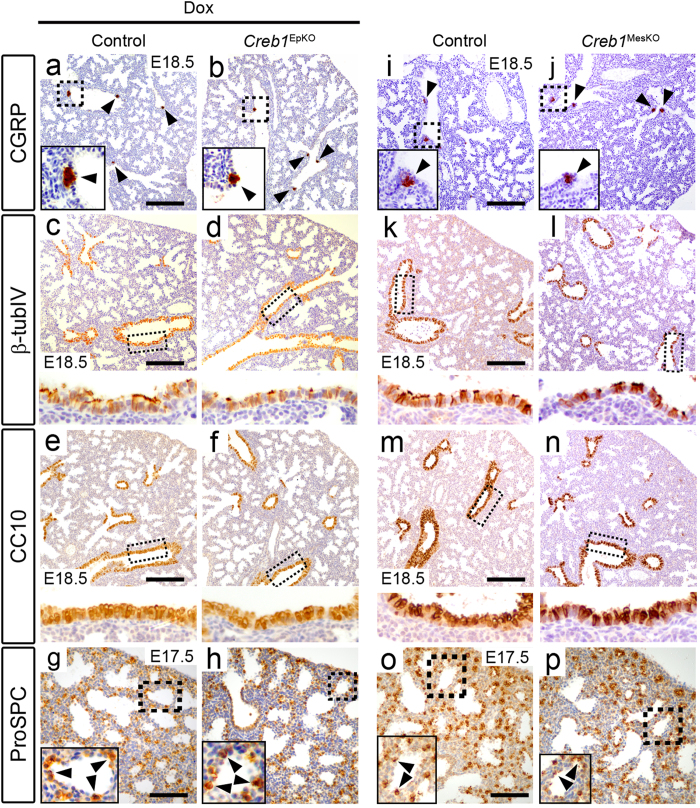
Normal epithelial differentiation in *Creb1*^EpKO^ lungs. Immunohistochemistry for the proximal epithelial markers CGRP (**a**,**b**, **i**,**j**), β-tubulin IV (**c**,**d**,**k**,**l**), and CC10 (**e**,**f**,**m**,**n**) in E18.5 *Creb1*^EpKO^ and *Creb1*^MesKO^ lungs. Immunohistochemistry for the distal epithelial (type-II AEC) marker ProSPC (**g**,**h**,**o**,**p**) in E17.5 *Creb1*^EpKO^ and E18.5 *Creb1*^MesKO^ lungs. No differences in expression or localization was observed for proximal or epithelial markers. Boxed areas are magnified either in insets (CGRP, ProSPC) or below the main image (β-tubulin IV, CC10), while arrowheads indicate marker-positive cells. All images are representative of at least three animals per genotype. Scale bars: *a-f*, *i-n*; 180 μm. *g,h,o,p*; 90 μm.

Although neonatal survival and fetal lung morphology appeared to be unaffected in *Creb1*^EpKO^ and *Creb1*^MesKO^ mice, we reasoned that abnormalities in lung epithelial cell differentiation may still be present based on our previous findings showing altered levels of proximal and distal epithelial cell markers in *Creb1*^*fl*−/−^ lungs^10^. We therefore used immunohistochemistry to investigate the differentiation of neuroendocrine, ciliated and secretory (Clara) cells in E18.5 *Creb1*^EpKO^ and *Creb1*^MesKO^ lungs using the proximal lung epithelial cell markers CGRP (also known as *Calca*), β-tubulin IV and CC10 (also known as *Scgb1a1*), respectively. However, we did not observe any difference in relative levels or spatial localization of these markers in *Creb1*^EpKO^ and *Creb1*^MesKO^ lungs (Fig. 3a–f,i–n). Distal epithelial cell differentiation also appeared unaffected in E18.5 *Creb1*^EpKO^ and *Creb1*^MesKO^ lungs as shown by immunohistochemistry for the type-II AEC marker, ProSPC (Fig. 3g,h,o,p). Using qPCR, we also examined gene expression of proximal and distal lung epithelial cell markers in E18.5 *Creb1*^EpKO^ and *Creb1*^MesKO^ lungs using qPCR. Consistent with our immunostaining results, no prominent alterations in mRNA levels were observed, though a reduction in levels for *FoxJ1* (in *Creb1*^EpKO^ lungs) and *Aqp5* (in *Creb1*^MesKO^ lungs) was noted (Supplementary Fig. S4).

### Loss of respiratory epithelial or mesenchymal Creb1 in fetal lung is not rescued by Crem or Atf1 compensation

**Figure 4:**
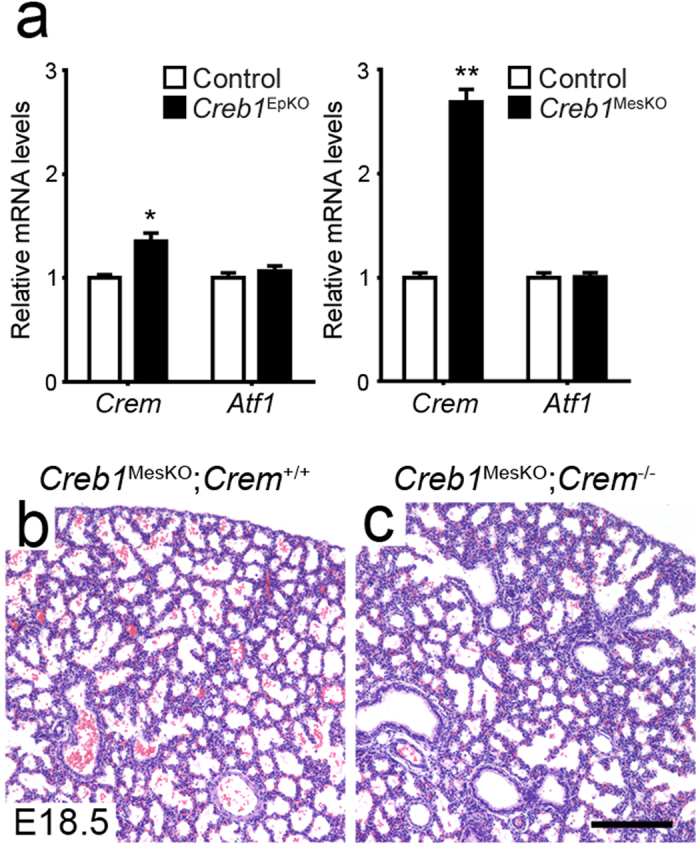
Lack of compensatory *Crem*/*Atf1* upregulation in *Creb1*^EpKO^ and *Creb1*MesKO lungs. (**a**) qPCR analysis of *Crem* and *Atf1* in E18.5 E18.5 *Creb1*^EpKO^ (*n* = 7) and *Creb1*^MesKO^ lungs (*n* = 6). (**b**,**c**) Haematoxylin and eosin-stained tissue sections from E18.5 control (*Creb1*^MesKO^; *Crem*^***+/+***^) and *Creb1*^MesKO^; *Crem*^−/−^ lungs. No major difference in lung morphology was observed. Error bars represent SEM. Single asterisk (*) indicates *p* < 0.05, while double asterisk (**) indicates *p* < 0.01. *White bars:* Controls, *Black bars:* Conditional Creb1 deletions. All images are representative of at least three animals per genotype. Scale bars: 180 μm.

To test for possible compensatory up-regulation by the Creb/Atf family members *Crem* and *Atf1*, we firstly examined mRNA levels for these factors in E18.5 *Creb1*^EpKO^ and *Creb1*^MesKO^ lungs using qPCR. We found no changes in *Atf1* mRNA levels in *Creb1*^EpKO^ or *Creb1*^MesKO^ lungs (Fig. 4a). However, *Crem* mRNA levels increased in *Creb1*^EpKO^ lungs (1.4 fold, *p* < 0.05), and a greater increase was observed in *Creb1*^MesKO^ lungs (2.7 fold, *p* < 0.001) (Fig. 4a). Despite this, immunohistochemical analysis using either an N-terminal or a C-terminal Crem antibody found no detectable levels of Crem protein in either control, *Creb1*^MesKO^ or *Creb1*^EpKO^ fetal lungs at E18.5 (Supplementary Fig. S5). Furthermore, we could not detect Crem protein in the embryonic lung at any developmental stage examined (E13.5, E15.5, PN6.5, Adult) (Supplementary Fig. S5). Normal Crem immunoreactivity (from both N- and C-terminal antibodies) however, was seen in round spermatid cell nuclei of the adult mouse testis, consistent with known sites of Crem expression^6^ (Supplementary Fig. S5). To further investigate potential Crem involvement in *Creb1*^MesKO^ lung development we bred *Creb1*^MesKO^ mice onto a *Crem*^+/−^ or *Crem*^−/−^ genetic background, and assessed fetal lung morphology at E18.5 as well as neonatal survival at 2 weeks postnatally. Fetal lungs from E18.5 *Creb1*^MesKO^; *Crem*^−/−^ mice appeared normally developed relative to *Creb1*^MesKO^; *Crem*^+/−^ controls (Fig. 4b,c), and immunohistochemical analysis of *Creb1*^MesKO^; *Crem*^−/−^ lungs did not exhibit any defect in epithelial differentiation (Supplementary Fig. S6). Interestingly however, numbers of *Creb1*^MesKO^; *Crem*^+/−^ and *Creb1*^MesKO^; *Crem*^−/−^ mice at 2 weeks was significantly lower than the expected 25% Mendelian frequency (Table 3). *Creb1*^MesKO^; *Crem*^−/−^ mice were never found, although due to extremely low progeny numbers and a high rate of maternal cannibalization there was insufficient statistical power to determine a causal link from this genotype to loss of survival (Supplementary Table S3). We did not attempt to generate *Creb1*^EpKO^; *Crem*^−/−^ mice due to several breeding limitations including *Crem*^−/−^ male sterility and also the inability to breed *Creb1*^*fl/fl*^ mice on a homozygote *SPCrtTA*^*tg/tg*^ background^21^. Together, these observations support the view that the normal lung phenotype in *Creb1*^EpKO^ and *Creb1*^MesKO^ mice is not due to compensatory up-regulation by Creb/Atf family members.

### Glucocorticoid hormone synthesis is unaffected in in *Creb1* ^*fl*−/−^ mice

**Figure 5:**
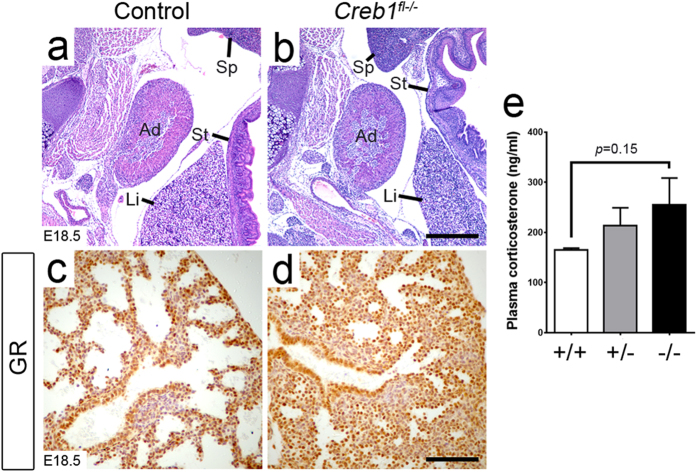
Loss of Creb1 does not impair GC signaling in the fetal lung. (**a**,**b**) Haematoxylin and eosin-stained tissue in transverse sections through lower thoracic vertebrae in E18.5 control and *Creb1*^*fl*−/−^ mice. Adrenal gland proportions appear comparable between control and *Creb1*^*fl*−/−^ mice. (**c**,**d**) Immunohistochemistry for GR in E18.5 control and *Creb1*^*fl*−/−^ lungs. (**e**) Measurement of plasma corticosterone in E18.5 *Creb1*^+/+^ (*white bars*), *Creb1*^+/−^ (*grey bars*) and *Creb1*^*fl*−/−^ (*black bars*) mice. Error bars represent SEM. All images are representative of at least three animals per genotype. *Ad*: adrenal gland, *Li*: liver, *Sp*: spleen, *St*: stomach. Scale bars: *a, b*: 360 μm, *c*, *d*: 90 μm.

As we had not observed any respiratory phenotype in *Creb1*^EpKO^ or *Creb1*^MesKO^ mice, we re-examined *Creb1*^*fl*−/−^ fetal mice and tested for disruption of the GC signaling pathway, which is required for late stage lung maturation via the GC Receptor (GR, *Nr3c1*)^22^. We examined relative adrenal gland proportions, plasma corticosterone levels as well as pulmonary GR expression in E18.5 control and *Creb1*^*fl*−/−^ fetal mice. However, transverse sections through lumbar vertebrae showed that adrenal glands in *Creb1*^*fl*−/−^ fetal mice appeared similar to controls (Fig. 5a,b). Corticosterone levels were slightly increased in *Creb1*^*fl*−/−^ fetal mice, though this did not reach statistical significance (Fig. 5e). Lastly, the level of GR immunoreactivity in *Creb1*^*fl*−/−^ fetal lungs was comparable to controls, and appeared to be localized primarily to the nucleus indicative of normal GC ligand-bound GR activation (Fig. 5c,d). Together, these results suggest that the respiratory phenotype in *Creb1*^*fl*−/−^ fetal mice is not caused by loss of GC signaling.

### Diaphragm and intercostal musculature development are unaffected in *Creb1* ^*fl*−/−^ mice

**Figure 6:**
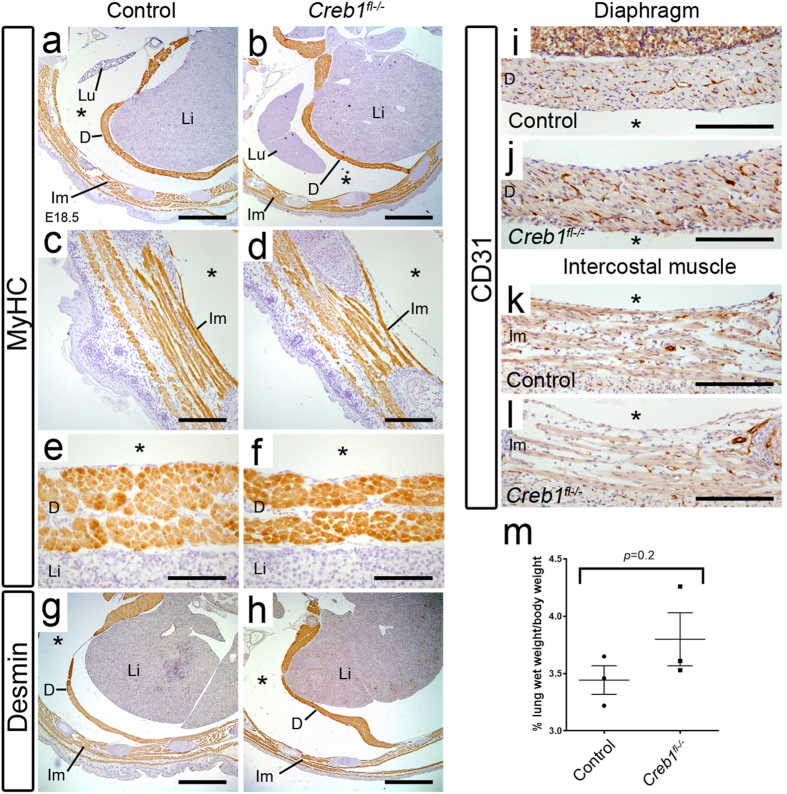
Diaphragm and intercostal musculature are normally developed in *Creb1*^*fl*−/−^ fetal mice. Immunohistochemistry for the muscle markers MyHC (**a**–**f**), desmin (**g**,**h**) and CD31 (**i**–**l**) in transverse sections through thoracic vertebrae in E18.5 control and *Creb1*^*fl*−/−^ fetal mice. Magnified images of MyHC-positive intercostal (**c**,**d**) and diaphragm (**e**,**f**) musculature. Intercostal and diaphragm musculature appear normally developed in *Creb1*^*fl*−/−^ fetal mice. (**m**) Scatterplot showing the percentage fetal lung wet weight/body weight in E18.5 control and *Creb1*^*fl*−/−^ littermates (*n* = 3). Error bars represent SEM. All images are representative of at least three animals per genotype. Scale bars: *a*,*b*,*g*,*h*; 900 μm, *c*,*d*,*i*-*l*; 180 μm, *e,f*; 90 μm. *Lu*: lung, *Li*: liver, *D*: diaphragm, *Im*: intercostal muscle, “*”indicates thoracic cavity.

We also re-examined *Creb1*^*fl*−/−^ fetal mice for defects in muscular elements known to be important for late stage lung development such as the diaphragm and intercostal muscle. We firstly inspected E18.5 *Creb1*^*fl*−/−^ fetal mice for possible diaphragm herniation, however careful examination of the thoracic cavity (in 3 separate knockout animals) did not reveal any noticeable defects in diaphragm muscle tissue. Secondly, we tested whether FBM-associated muscle fibres were properly formed in *Creb1*^*fl*−/−^ fetal mice at E18.5 using immunohistochemistry for the mature muscle marker myosin heavy chain (MyHC). In transverse sections through thoracic vertebrae, we noticed no difference in MyHC-positive diaphragm or skeletal intercostal muscle fibres in *Creb1*^*fl*−/−^ mice compared with controls (Fig. 6a–f). Another Creb1-regulated structural muscle protein, desmin^23^, similarly appeared at normal levels in all skeletal and diaphragm musculature examined in E18.5 *Creb1*^*fl*−/−^ fetal mice (Fig. 6g,h). In addition, CD31 immunohistochemistry showed that these muscular elements were properly vascularized (Fig. 6i–l). Lastly, we tested for evidence of lung hypoplasia in E18.5 *Creb1*^*fl*−/−^ mice via analysis of lung wet weight/body weight ratios (Fig. 6m). No difference was detected between *Creb1*^*fl*−/−^ (3.8 ± 0.2%, *n* = 3) and *Creb1*^*fl*+/−^ littermate controls (3.4 ± 0.1%, *n* = 3). Thus, it is unlikely that the respiratory phenotype in *Creb1*^*fl*−/−^ mice is due to loss of in utero FBM-associated muscular activity.

## Discussion

In this study we initially show that conditional *Creb1* deletion in the respiratory epithelium, endothelium or mesenchyme surprisingly has no major impact on lung sacculation, suggesting that lung-localized Creb1 has no essential role in mammalian lung development. While Creb1 is widely expressed in the developing lung, the primary site of Creb1 transcriptional function is believed to be the distal epithelium, based on the expression of Ser133 pCreb1 in the pseudoglandular phase^10^,^13^. Our Creb1 immunohistochemistry in *Creb1*^EpKO^ mice showed that a subset of epithelial cells, primarily from proximal regions, often escaped deletion. It is therefore possible that sufficient Creb1 activity allowed normal lung development to proceed in *Creb1*^EpKO^ mice. While it is expected that a small subset of proximal epithelial cells will not undergo *Creb1* deletion due to lack of *SPCrtTA* transgene activation (such as the neuroepithelial lineage^24^), it is also conceivable that the *Creb*^*fl*^ locus is somewhat resistant to Cre-mediated recombination. This latter possibility appears more likely given that we observe no expansion of neuroepithelial (or other) proximal epithelial lineages in *Creb1*^EpKO^ lungs. Nevertheless, our finding of largely complete *Creb1* deletion in the distal epithelium of *Creb1*^EpKO^ lungs argue against a normal phenotype in these mice resulting from residual epithelial Creb1 signaling. On the other hand, strong Ser133 pCreb1 expression in mesenchymal cells during the saccular phase also argues for a role for Creb1 in the mesenchyme^10^, yet while *Dermo1*^*Cre*^-mediated deletion appeared mostly complete in *Creb1*^MesKO^ lungs, we also did not observe any respiratory phenotype in these mice.

Interestingly, mice with a Ser133 to Alanine residue mutation (*Creb1* Ser133A) in the *Creb1* gene are viable and healthy, albeit born at a reduced Mendelian frequency when bred on a C57BL/6J background^25^. This suggests that distal epithelial Ser133 pCreb1 expression is not an indicator of important Creb1 activity in the lung, and/or that a partial functional redundancy exists between other phosphorylation sites for Creb1 transcriptional activation and normal embryonic development. It would be valuable to generate mice with compound Creb1 phosphorylation site mutations to pursue this hypothesis. In addition, it would be worthwhile to generate compound lung epithelial/mesenchymal Creb1 mouse mutants to investigate the potential combined requirement of Creb1 in these germ layers for normal lung development.

We next investigated several alternate mechanisms in order to explain the severe lung phenotype observed in total *Creb1* mouse mutants. Firstly, we provide evidence that the lack of a respiratory phenotype in conditional *Creb1* deletions is not caused by compensatory activities of Crem or Atf1. This functional redundancy within the Creb/Atf1 subfamily is believed to occur via up-regulation of these factors’ expression in the absence of Creb1^8^,^26^,^27^. Our investigation shows that while upregulation of *Crem*, and not *Atf1*, gene expression takes place in both E18.5 *Creb1*^EpKO^ and *Creb1*^MesKO^ fetal lungs, the lack of Crem immunoreactivity in both controls and conditional knockout lungs indicates that Crem protein is neither synthesized in the wildtype fetal lung, nor upregulated to detectable levels in the absence of Creb1. Although we did not test all *Crem* isoforms in our qPCR and immunohistochemcial analyses, more compelling is our finding that lungs of *Creb1*^MesKO^ mice, which showed a higher upregulation of *Crem* than *Creb1*^EpKO^ lungs, displayed no lung phenotype even when bred on a *Crem* deficient genetic background. It is therefore possible that in most tissues, *Crem* mRNA upregulation in the absence of Creb1 has little or no functional outcome, with the exception of perhaps the brain where Crem compensation protects against neuronal cell death^11^. Interestingly however, our finding of reduced neonatal survival in *Creb1*^MesKO^; *Crem*^+/−^ and *Creb1*^MesKO^; *Crem*^−/−^ mice point to a compensatory role for Crem in Creb1-deficient mesodermal tissues during early postnatal life. This phenotype would be interesting to investigate, however given the wide range of non-respiratory organs also targeted by the *Dermo1*^*Cre*^ allele including bone^16^, kidney^28^ and heart^29^, isolating the affected system(s) may be a challenging task.

Tracing the origin of respiratory defects in mouse mutants can be difficult given that a loss of gene function in a separate tissue can potentially impair lung development via an indirect (or non-autonomous) mechanism. For example, mice lacking the myogenic factor Myogenin (*Myog*) exhibit defective lung sacculation due to poorly developed diaphragm muscle^30^, though *Myog* is not expressed in the lung. Similarly, loss of the skeletal muscle-specific factor *Myf5*, also not expressed in the lung, prevents normal lung sacculation^31^. More difficult to interpret are lung phenotypes from total mouse mutants in which the deleted gene is expressed in multiple tissues including lung, yet other musculoskeletal defects are also present which may contribute or represent the sole cause of the lung phenotype. For example, the insulin-like growth factor-1 (*Igf1r*) is widely expressed during embryonic development, and *Igf1r* total knockout mice exhibit both fetal lung and diaphragmatic defects^32^.

Advantageously, the multiple systems targeted by the *Dermo1*^*Cre*^ allele also help to explore whether certain non-respiratory mechanisms potentially underpin the *Creb1*^−/−^ lung phenotype. For example, it could be argued that impaired development of the ribcage or vertebrae may prevent normal expansion of the underlying lung or provide inadequate structural support to respiratory muscles, thus arresting lung development prior to sacculation^33^. This suggestion has significant merit given the vertebral fusions occasionally observed in *Creb1*^−/−^ mice^34^ but is also supported by previous findings in mice which utilize tissue-specific overexpression of a dominant negative Creb1 transgene known as A-CREB, which specifically blocks DNA binding of the Creb/Atf1 subfamily^35^. A-CREB overexpression in chondrocytes results in a reduced rib-cage circumference and neonatal lethality^36^, while mice overexpressing A-CREB in the mesoderm also die at birth, presumably due to respiratory failure and exhibit profound ribcage, as well as other skeletal defects^34^. Conversely, we did not observe any lung or skeletal phenotype in *Creb1*^MesKO^ or *Creb1*^MesKO^; *Crem*^−/−^ fetal mice, although a lack of Creb1 expression in ribcage chondrocytes due to *Dermo1*^*Cre*^-mediated deletion was apparent in these mutants. We speculate that these discrepancies are partially due to differences in promoter-specific *Dermo1*^*Cre*^ versus A-CREB expression in mesodermal cell lineages, although it is also conceivable that Atf1 can compensate for lack of Creb1/Crem for correct skeletal development. Another possibility is that the lack of CRE occupancy in A-CREB mutants leads to aberrant promoter binding by other factors which may produce off-target developmental outcomes via abnormal target gene regulation. We therefore suggest a level of caution when interpreting the phenotype in A-CREB mouse mutants.

We also investigated, but excluded, the possibility that the *Creb1*^−/−^ lung phenotype is secondary to muscular defects. Late stage lung development is known to be critically dependent on mechanical influences from *in utero* ‘fetal breathing movements’ (FBMs), which are driven by contractile activities of the diaphragm and skeletal intercostal musculature. Absence of skeletal muscles results in lung hypoplasia during late *in utero* development as seen in compound knockout mice which lack both of the myotomal factors *Myf5* and *MyoD*^37^. Abnormalities in diaphragm development can similarly cause lung hypoplasia, most commonly due to diaphragmatic herniation whereby abdominal contents invade the thoracic space and prevent fetal lung expansion^38^. Intriguingly, a previous study using *Creb1*^−/−^ mice found PKA-Creb1 signaling to be required for important myotomal factors including *Pax3*, *MyoD* and *Myf5* during early development^39^. However, our MyHC immunohistochemistry show that *Creb1*^*fl*−/−^ mice do not exhibit any defects in FBM-associated musculature that would be consistent with a loss of saccular lung development. Furthermore, previous and current findings from our group have not found any evidence of lung hypoplasia in *Creb1*^*fl*−/−^ mice, which would be expected in the event of impaired FBM-associated musculature. For example, in our current study the lung wet weight/body weight ratios in *Creb1*^*fl*−/−^ fetal mice were comparable to controls, though this is normally reduced in lung hypoplasia. Additionally, our previous work in E18.5 *Creb1*^*fl*−/−^ lungs showed no significant difference in cell apoptosis and a slightly higher rate of cell proliferation^10^, though these are normally increased and reduced, respectively, in lung hypoplasia.

Another potential mechanism explored was the possibility that global loss of Creb1 disrupts components of the GC-GR signaling pathway, which is required for late stage lung maturation. On one hand, insufficient levels of corticosterone (the active rodent GC ligand) prevent GR transcriptional signaling as seen in corticotrophin releasing hormone (*Crh*) knockout mice^40^. On the other hand, GR mouse mutants exhibit elevated corticosterone levels secondary to marked adrenal hypertrophy^22^. Interestingly, Creb1 has a recognized role in promoting gene expression of *Crh*^41^, supporting a role for Creb1 in GC production. However, free corticosterone levels appeared relatively unchanged in *Creb1*^*fl*−/−^ mice, consistent with normally-sized adrenal glands. In addition, our finding of nuclear GR localization in *Creb1*^*fl*−/−^ lungs implies that sufficient GC ligand is available to promote cytoplasmic to nuclear GR translocation. The normal levels of corticosterone in *Creb1*^*fl*−/−^ mice are perhaps unsurprising given that a fetal loss of GCs in various GC signaling deficient mouse mutants can be compensated for by a maternal transfer of corticosterone across the placenta^42^. Therefore, while our results do not preclude an absence of *Crh* expression in *Creb1*^*fl*−/−^ mice, this is unlikely to be the cause of the *Creb1*^*fl*−/−^ respiratory phenotype. Normal GR expression in *Creb1*^*fl*−/−^ lungs also suggests Creb1 is not required for lung GR expression.

In summary, we find that the requirement of Creb1 for normal mammalian lung development is not dependent upon its expression in the respiratory epithelium or mesenchyme. Furthermore, we show that this requirement is not indirectly due to known or suspected Creb1 involvement in musculoskeletal development, nor the GC signaling pathway. Therefore, the underlying cause behind the respiratory phenotype in total *Creb1* mouse mutants remains unknown. One further possibility not explored in this study is whether the recognized involvement of Creb1 in the peripheral nervous system^43^ indirectly stimulates lung sacculation via innervation of FBM-associated muscles. However, while innervation of muscular elements such as the diaphragm is known to be required for lung function at birth^44^, it is uncertain whether it is also essential for the saccular process of lung development. Conditional Creb1 deletion in a wider range of neuronal cell lineages would therefore prove a valuable exercise to address this potential cause of the lung phenotype observed in *Creb1*^*fl*−/−^ mice.

## Methods

### Mouse models

SPCrtTA and TetO-cre mice were purchased from the Jackson Laboratory (Bar Harbor, Maine, USA). Dermo1-cre, CMV-cre and Tie2-cre mice were generously provided by Dr. Brandon Wainwright (University of Queensland, Brisbane, Australia), Dr. Christina Mitchell (Monash University, Melbourne, Australia) and Dr. Jinhua Li (Monash University) respectively. All mouse strains were maintained on a C57BL/6 background. To generate the *Creb1*^EpKO^ mice, SPCrtTA and TetO-cre lines were both bred to *Creb1*^*fl/fl*^ mice to produce transgenic (tg) *Creb1*^*fl/fl*^;*SPCrtTA*^*tg/*−^ and *Creb1*^*fl/fl*^;*TetO-cre*^*tg/*−^ mice, respectively. These two lines were then time-mated, and rodent food was altered at E6.5 till E14.5 to contain doxycycline (600 mg/kg, Specialty Feeds, WA, Australia) to induce cre expression in pups bearing all three transgenic alleles (*Creb1*^EpKO^). Genotyping primer sequences are shown in Supplementary Table S1. All animal experimentation was approved and carried out according to the guidelines established by the School of Biomedical Sciences Animal Ethics Committee, Monash University (Ethics No. 2011/147).

### Histology, Immunohistochemistry and Lung Tissue morphometry

Whole fetal mouse torsos were immersion-fixed in 4% paraformaldehyde (PFA) overnight at 4 °C with agitation then embedded in paraffin. 5μm sections were cut and mounted on slides, then used for histological or immunostaining analyses according to a standard protocol as previously described^10^. Lung tissue morphometry was performed according to previously published methods^45^. Primary antibodies used are shown in Supplementary Table S2.

### RNA Extraction, cDNA synthesis and quantitative PCR

Total RNA and cDNA was obtained from fetal lungs using TRIzol reagent (Invitrogen, Carlsbad, CA) and M-MLV Reverse Transcriptase (Promega, Madison WI), respectively, as per the manufacturer’s instructions. The integrity of 28S and 18S rRNA from total RNA was always assessed on agarose gels prior to further analysis. qPCR analysis was performed in triplicate for each biological replicate using at least two housekeeping genes per analysis: 18S rRNA and Rps29. Other qPCR primers were designed to include at least one oligo overlapping an exon-exon boundary, using the web-based software Primer3^46^. Primer efficiency was then calculated using a standard curve and fetal lung cDNA template. Sequences for these primers can be found in a previous publication^10^. Cycling was performed on a CFX384 Touch™ Real-Time PCR Detection System (Bio-Rad, Richmond, CA). Relative differential expression was then determined using CFX Manager™ software (Bio-Rad).

### Corticosterone measurement

Trunk blood (5–10 μl) was obtained from fetal wildtype and *Creb1*^*fl*−/−^ mice and assayed for free corticosterone levels using a Corticosterone Double Antibody RIA Kit (MP Biomedicals, Solon, OH), as per the manufacturer’s instructions. Radioactivity was measured using a 1470 Perkin Elmer automated gamma counter (Perkin Elmer, Waltham, MA).

### Statistical Analysis

GraphPad Prism software was used to analyze the results of all experiments with a statistical significance set at *p* < 0.05. Statistically significant deviations in neonatal mouse survival at two weeks of age was calculated using a goodness-of-fit test.

## Additional Information

**How to cite this article**: Antony, N. *et al.* Creb1 regulates late stage mammalian lung development via respiratory epithelial and mesenchymal-independent mechanisms. *Sci. Rep.* **6**, 25569; doi: 10.1038/srep25569 (2016).

## Supplementary Material

Supplementary Information

## Figures and Tables

**Table 1:** Analysis of survival at two weeks of age in *Creb1* ^EpKO^ mice.

Cross	Litters	Pups	Average litter size	Creb1^fl/fl^	Creb1^fl/fl^; TetO-cre^*tg/*−^	Creb1^fl/fl^; SPCrtTA^tg/−^	Creb1^fl/fl^; SPCrtTA^*tg/*−^; TetO-cre^*tg/*−^	Loss of Creb1^EpKO^ pups
Creb1^fl/fl^; TetO-cre^*tg/*−^ × Creb1^fl/fl^; SPCrtTA^*tg/*−^ +Doxycycline	8	50	6.25	20 (12.5)	10 (12.5)	11 (12.5)	9 (12.5)	Non-significant

**Table 2:** Analysis of survival at two weeks of age in *Creb1* ^MesKO^ mice.

Cross	Litters	Pups	Average litter size	*Creb1*^*fl/f*^; *Dermo1*^+/+^	*Creb1*^*fl/fl*^; *Dermo1*^*Cre/*+^	Loss of *Creb1*^MesKO^ pups
*Creb1*^*fl/fl*^; *Dermo1*^+/+^; × *Creb1*^*fl/fl*^; *Dermo1*^*Cre/*+^	11	65	5.9	40 (32.5)	24 (32.5)	Non-significant

**Table 3:** Analysis of survival at two weeks of age in *Creb1* ^MesKO^; *Crem* ^+/−^ mice.

Cross	Litters	Pups	Average litter size	Creb1^fl/fl^; Dermo1^+/+^; Crem^+/+^	Creb1^fl/fl^; Dermo1^+/+^; Crem^+/−^	Creb1^fl/fl^; Dermo1^*Cre/*+^; Crem^+/+^	Creb1^fl/fl^; Dermo1^*Cre/*+^; Crem^+/−^	Loss of Creb1^MesKO^; Crem^+/−^ pups
Creb1^fl/fl^; Dermo1^*Cre/*+^; Crem^+/−^ × Creb1^fl/fl^; Dermo1^+/+^; Crem^+/+^	23	129	5.6	45 (28.5)	38 (28.5)	33 (28.5)	13 (28.5)	Significant loss p = 0.0005

The genotype of the progeny was determined as described in *Materials and Methods* at two weeks of age. The expected number of each genotype is indicated in brackets, and was calculated according to a predicted Mendelian allele inheritance ratio. Statistically significant deviations from the expected frequency was determined using a goodness-of-fit test with significance set a *p* < 0.05.
