# Supplementary material for: Creb1 regulates late stage mammalian lung development via respiratory epithelial and mesenchymal-independent mechanisms
Source: Sci Rep. 2016 May 6;6:25569. doi: 10.1038/srep25569 (PMC4858709; doi:10.1038/srep25569)
Supplement: Supplementary Information [file srep25569-s1.pdf]

Creb1 regulates late stage mammalian lung development via respiratory epithelial and mesenchymal-independent mechanisms

Antony N, McDougall R.A, Mantamadiotis T, Cole T.J, Bird A.D

Supplementary Information

Supplementary Figure S1: Creb1 deletion in *Creb1*^{EpKO} and *Creb1*^{EndoKO} lungs, and *Creb1*^{MesKO} chondrocytes

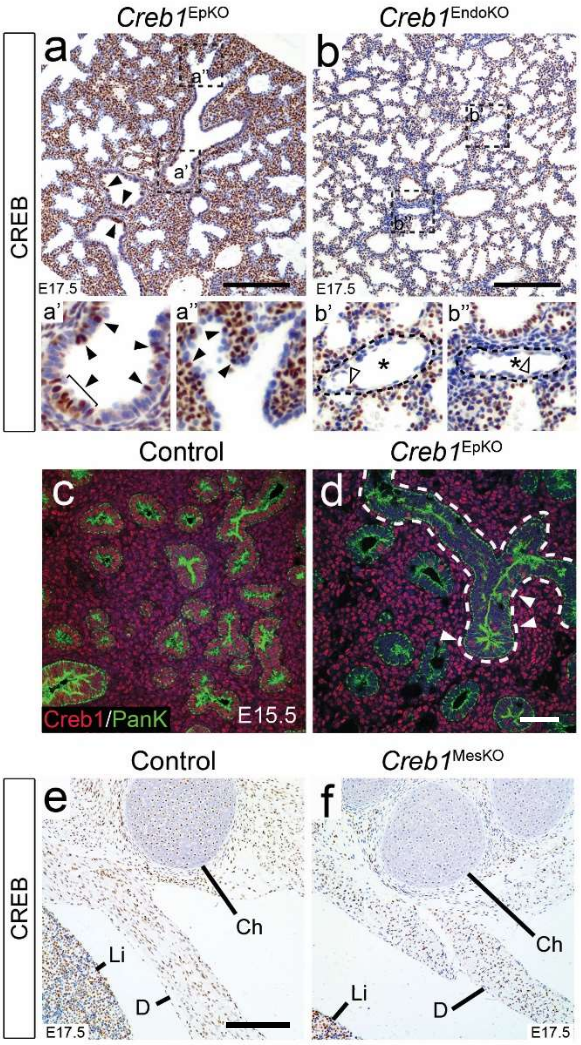

(a) Immunohistochemistry for Creb1 showing variable Creb1 deletion within the proximal epithelium of E17.5 *Creb1*^{EpKO} lungs. Boxed areas within the main image are magnified below to show proximal (a') and distal (a'') regions of epithelium, respectively. Arrowheads indicate Creb1-positive cells and cell clusters, the latter shown in a bracketed region in (a').

(b) Immunohistochemistry for Creb1 showing efficient Creb1 deletion in endothelial cells of E17.5 *Creb1*^{EndoKO} lungs. Boxed areas within the main image are magnified below to show a pulmonary vein (b') and artery (b''), respectively. The blood vessel lumen is indicated by a "*" and rare Creb1-positive cells are shown by open arrowheads. (c, d) Double-label immunofluorescence for Creb1 and the epithelial marker pan-keratin (PanK) in E15.5 *Creb1*^{EpKO} lungs. Dashed lines in (d) depict a region of proximal-distal epithelium with arrowheads indicating Creb1-positive cells mostly found in the proximal epithelium. (e, f) Immunohistochemistry for Creb1 showing Creb1 deletion in E17.5 *Creb1*^{MesKO} ribcage chondrocytes. Sections were cut in the sagittal plane. *Li*: liver, *D*: diaphragm, *Ch*: chondrocytes in ribcage. Scale bars: a, b, e, f; 180 µm, c, d; 50 µm.

Supplementary Figure S2: Normal tissue cellularity in lungs of fetal *Creb1*^{EpKO} mice

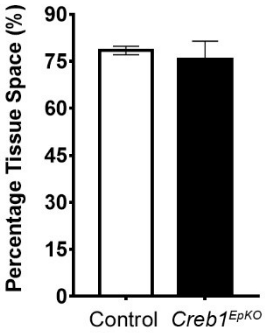

Tissue-to-airspace ratio quantification based on lung sections from E17.5 control (*n*=5) and *Creb1*^{EpKO} lungs (*n*=7). *White bars:* Controls, *Black bars:* Conditional Creb1 deletions.

Supplementary Figure S3: Normal lung development in early postnatal *Creb1*^{EpKO} and *Creb1*^{MesKO} mice

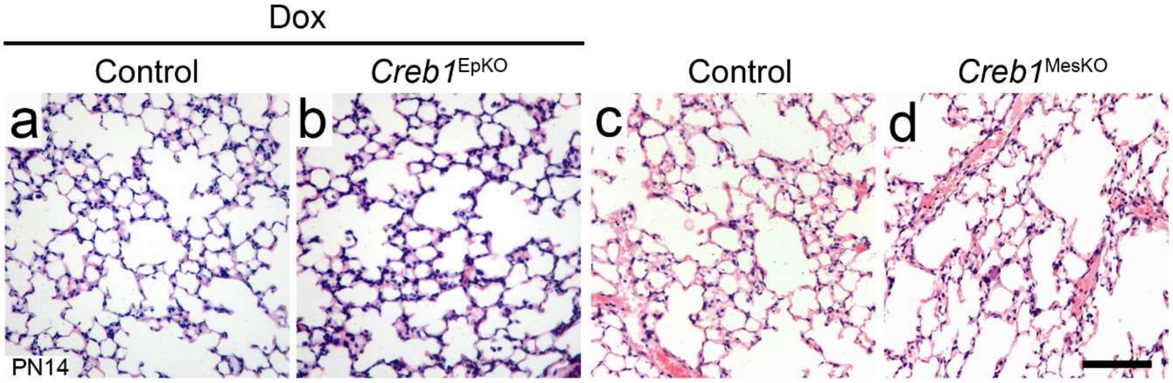

Haematoxylin and eosin-stained tissue sections from PN14 Dox-treated control (a) *Creb1*^{EpKO} (b), non-dox treated control (c) and *Creb1*^{MesKO} lungs (d). All images are representative of at least three animals per genotype. Scale bar: 180 µm.

Supplementary Figure S4: Unchanged epithelial marker expression in E18.5 *Creb1*^{EpKO} and *Creb1*^{MesKO} lungs

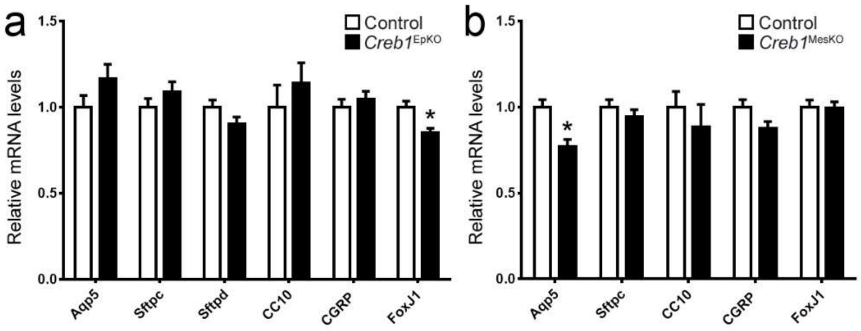

qPCR analysis of proximal and distal epithelial cell markers in (a) E18.5 *Creb1*^{EpKO} (*n*=7) and (b) *Creb1*^{MesKO} lungs (*n*=6). Error bars represent SEM. Single asterisk (*) indicates p<0.05. *White bars:* Controls, *Black bars:* Conditional Creb1 deletions.

Supplementary Figure S5: Crem protein is not upregulated in E18.5 *Creb1*$^{\mathbf{EpKO}}$ and *Creb1*$^{\mathbf{MesKO}}$ lungs

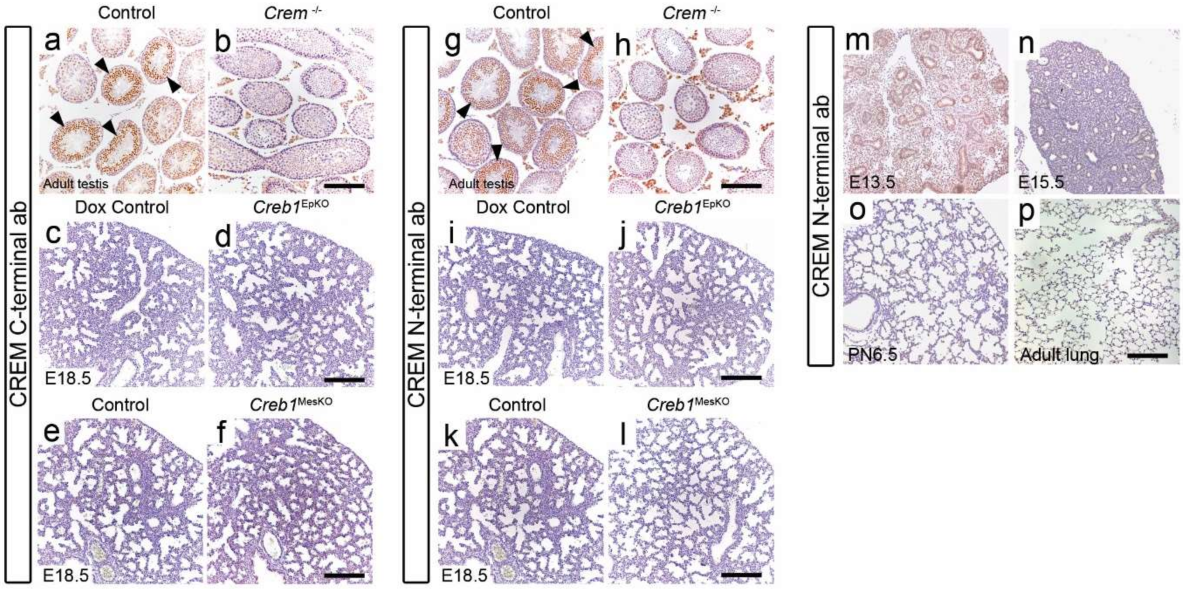

Immunohistochemistry for Crem in wildtype mouse adult testis and lungs from conditional *Creb1* deletions. Immunohistochemistry for C-terminal Crem in wildtype adult mouse testis (a), *Crem* $^{-/-}$ adult mouse testis (b), Dox-treated control E18.5 lung (c), E18.5 *Creb1*^{EpKO} lung (d), E18.5 non-dox treated control lung (e) and E18.5 *Creb1*^{MesKO} lungs (f). C-terminal Crem expression was observed only in wildtype testes tissue. Immunohistochemistry for N-terminal Crem in wildtype adult mouse testis (g), *Crem* $^{-/-}$ adult mouse testis (h), Dox-treated control E18.5 lung (i), E18.5 *Creb1*^{EpKO} lung (j), E18.5 non-dox treated control lung (k) and E18.5 *Creb1*^{MesKO} lungs (l). N-terminal Crem expression was observed only in wildtype testes tissue. Immunohistochemistry for N-terminal Crem in lungs from wildtype E13.5 (m), E15.5 (n), PN6.5 (o), Adult (p). N-terminal Crem expression was not detected in lung from any developmental stage. Moderate non-specific C- and N-terminal Crem staining was observed in Leydig cells in (a, b, g, h). Arrowheads indicate Crem-positive nuclei in round spermatids in (a, g). All images are representative of at least three animals per genotype. Scale bars: 180 μm.

Supplementary Figure S6: Normal epithelial differentiation in *Creb1*^{MesKO}; *Crem*$^{-/-}$ lungs

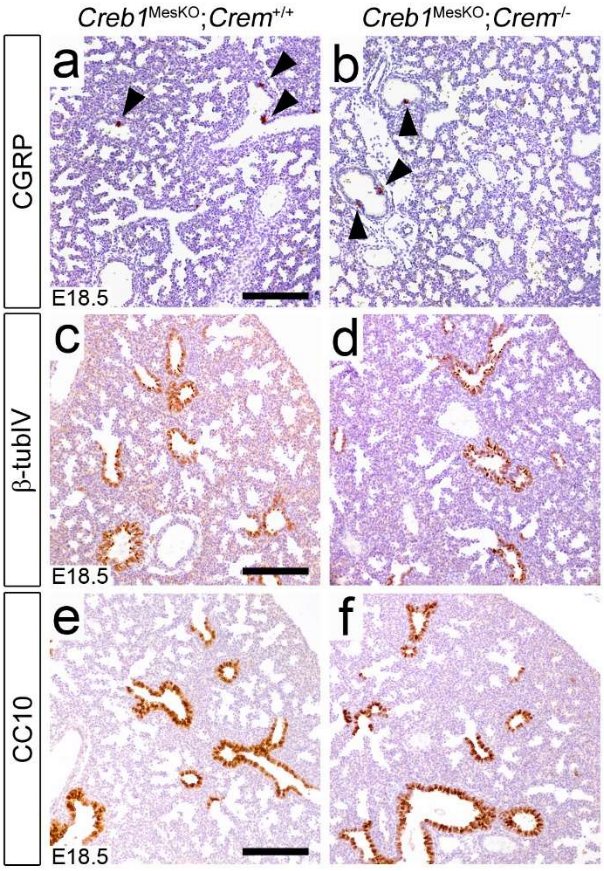

Immunohistochemistry for the proximal epithelial markers CGRP (a,b), β-tubulin IV (c, d), and CC10 (e, f) in E18.5 control and *Creb1*^{MesKO}; *Crem*$^{-/-}$ lungs. No differences in expression or localization was observed for proximal or epithelial markers. Arrowheads indicate CGRP-positive cells. All images are representative of at least three animals per genotype. Scale bars: 180 μm.

Supplementary Table S1: Genotyping primers

**Allele**	**Sequence**
Creb1^{fl}	forward: 5' TATGTAAAGCAAGGGAAGATACTG 3'<br>internal: 5' TAGACATACTTGACCCATAGCATT 3'<br>reverse: 5 ' GTGATTCCAGCACTCACTCC 3'
SPCrtTA^{tg}	forward: 5’ GACACATATAAGACCCTGGTCA3’<br>reverse: 5’ AAAATCTTGCCAGCTTTCCCC 3’
TetO-cre^{tg}	forward: 5’ TGCCACGACCAAGTGACAGCAATG 3’<br>reverse: 5’ AGAGACGGAAATCCATCGCTCG 3’
Crem	forward: 5' TGGATTGTGCTGGGAGGTTGTTC 3'<br>internal: 5’ CGCCATTCGCCATTCAGGCTG 3
Dermo1^{Cre}	forward: 5’ AACTTCCTCTCCCGGAGACC 3’<br>internal: 5’ TGCCTCTCCAGCTCTTCCTC 3’<br>reverse: 5’ CCGGTTATTCAACTTGCACC 3’

Supplementary Table S2: Primary antibodies

**Antibody**	**Manufacturer**	**Catalogue number**	**Dilution**
Creb1	Cell Signaling	9197	1:800
Ser133-pCreb1	Cell Signaling	9198	1:50
CD31	Abcam	ab28364	1:50
CREM (N-terminal)	Santa Cruz	sc-390425	1:2000
CREM (C-terminal)	Genetex	GTX114146	1:800
CGRP	Sigma-Aldrich	C8198	1:6000
B-tubulin IV	Abcam	ab11315	1:500
CC10	Santa Cruz	sc-9772	1:200
ProSPC	Millipore	AB3786	1:2000
MyHC	DSHB	A4.1025	1:10
Desmin	Abcam	ab86083	1:100
GR	Santa Cruz	sc-1004	1:1000
pan-keratin	Sigma-Aldrich	P2871	IF

Supplementary Table S3: Analysis of survival at two weeks of age in *Creb1*^{MesKO}; *Crem*$^{-/-}$ mice

**Cross**	**Litters**	**Pups**	**Average litter size**	***Creb1*$^{fl/fl}$; *Dermo1*$^{+/+}$; *Crem*$^{+/-}$**	***Creb1*$^{fl/fl}$; *Dermo1*$^{+/+}$; *Crem*$^{-/-}$**	***Creb1*$^{fl/fl}$; *Dermo1*$^{cre/+}$; *Crem*$^{+/-}$**	***Creb1*$^{fl/fl}$; *Dermo1*$^{cre/+}$; *Crem*$^{-/-}$**
♂*Creb1*$^{fl/fl}$; *Dermo1*$^{Cre/+}$; *Crem*$^{+/-}$ × ♀*Creb1*$^{fl/fl}$; *Dermo1*$^{+/+}$; *Crem*$^{-/-}$	3	14	4.7	6 (1.5)	5 (1.5)	3 (1.5)	0 (1.5)

The genotype of the progeny was determined as described in *Materials and Methods* at two weeks of age. The expected number of each genotype is indicated in brackets, and was calculated according to a predicted Mendelian allele inheritance ratio.
